# Supplementary material for: Seasonal variations in household food security and consumption affect women’s nutritional status in rural South Ethiopia
Source: PLOS Glob Public Health. 2024 Aug 20;4(8):e0003294. doi: 10.1371/journal.pgph.0003294 (PMC11335107; doi:10.1371/journal.pgph.0003294)
Supplement: S1 Table — This table provides the socio-demographic information of 1,089 women participants from 894 households. The data are presented as count (n) and percentage (%). The abbreviation “N” refers to the total number of participants. (DOCX) [file pgph.0003294.s001.docx]

S1 Table: Socio-demographic characteristics of 15 to 49 years old women in South Ethiopia, June 2021 (N=1,089)

| **Variables** | **Frequency n (%)** |
| --- | --- |
| **Age group** |  |
| 15-19 | 135 (12.4) |
| 20-24 | 108 (9.9) |
| 25-29 | 289 (26.5) |
| 30-34 | 219 (20.1) |
| 35-39 | 208 (19.1) |
| >=40 | 130 (11.9) |
| **Relationship** |  |
| Household head | 31 (2.8) |
| Wife | 866 (79.5) |
| Child | 190 (17.5) |
| Other (relatives and maid) | 2 (0.2) |
| **Marital status** |  |
| Married | 865 (79.4) |
| Single | 191 (17.6) |
| Other (Divorced and Widowed) | 33 (3) |
| **Educational status** |  |
| Couldn't read and write | 556 (51.1) |
| Read and write | 72 (6.6) |
| Primary | 381 (35) |
| Secondary and above | 80 (7.4) |
| **Occupational status** |  |
| Farmer | 778 (71.5) |
| Student | 205 (18.8) |
| Trader | 69 (6.3) |
| Other (employed, dependent, daily laborer) | 36 (3.3) |
| **Ethnicity** |  |
| Sidama | 1083 (99.5) |
| Others (Wolaita and Amhara) | 6 (0.5) |
| **Religion** |  |
| Protestant | 927 (85.1) |
| Muslim | 45 (4.1) |
| Catholic | 61 (5.6) |
| Others | 56 (5.1) |
| **Family size** |  |
| < =5 | 541 (49.7) |
| >5 | 548 (49.3) |

N.B: This table provides the socio-demographic information of 1,089 women study participants from 894 households. The data are presented as count (n) and percentage (%). The abbreviation “N” refers to the total number of participants.
