# Supplementary material for: Seasonal variations in household food security and consumption affect women’s nutritional status in rural South Ethiopia
Source: PLOS Glob Public Health. 2024 Aug 20;4(8):e0003294. doi: 10.1371/journal.pgph.0003294 (PMC11335107; doi:10.1371/journal.pgph.0003294)
Supplement: S2 Table — This table presents the socio-demographic information of the household heads where the study participants reside. The data are presented as count (n) and percentage (%). (DOCX) [file pgph.0003294.s002.docx]

S2 Table: Socio-demographic characteristics of household heads, South Ethiopia, June 2021 (N =894)

| **Variables** | **Frequency n (%)** |
| --- | --- |
| **Age category in years** |  |
| < 25 | 3 (0.3) |
| 25-29 | 100 (11.2) |
| 30-34 | 216 (24.2) |
| 35-39 | 194 (21.7) |
| 40-45 | 275 (30.8) |
| >45 | 106 (11.9) |
| **Sex** |  |
| Male | 861 (96.3) |
| Female | 33 (3.7) |
| **Marital status** |  |
| Married | 862 (96.4) |
| Others (Divorced and Widowed) | 32 (3.6) |
| **Educational status** |  |
| No formal education | 537 (60.1) |
| Primary | 244 (27.3) |
| Secondary | 99 (11.1) |
| Above secondary | 14 (1.6) |
| **Occupational status** |  |
| Farmer | 581 (65) |
| Employed | 25 (2.8) |
| Daily laborer | 72 (8.1) |
| Trader | 125 (14.0) |
| Student | 59 (6.6) |
| Others (no job, housemaid) | 32 (3.6) |
| **Ethnicity** |  |
| Sidama | 889 (99.4) |
| Others (Wolaita and Amhara) | 5 (0.6) |
| **Religion** |  |
| Protestant | 761 (85.1) |
| Muslim | 39 (4.4) |
| Catholic | 49 (5.5) |
| Others | 45 (5.0) |

N.B: This table presents the socio-demographic information of the household heads where the study participants reside. The data are presented as count (n) and percentage (%). The abbreviation “N” refers to the total number of participants.
