# Supplementary material for: Seasonal variations in household food security and consumption affect women’s nutritional status in rural South Ethiopia
Source: PLOS Glob Public Health. 2024 Aug 20;4(8):e0003294. doi: 10.1371/journal.pgph.0003294 (PMC11335107; doi:10.1371/journal.pgph.0003294)
Supplement: Attachment — Submitted filename: letter of rebutal.docx [file pgph.0003294.s003.docx]

Bethelhem Mezgebe , Taye Gari, Mehretu Belayneh, Bernt Lindtjørn

School of Public Health, College of Medicine and Health Sciences, Hawassa University, Hawassa, Ethiopia

[bethelhem_mezgebe@yahoo.com](mailto:bethelhem_mezgebe@yahoo.com)

[tayegari@ymail.com](mailto:tayegari@ymail.com)

[belaynehdinage@gmail.com](mailto:belaynehdinage@gmail.com)

bernt.lindtjorn@uib.no

Date 15/02/24

Subject: Rebuttal Letter for Re-submission of Rejected Manuscript

Dear Editor,

I hope this letter finds you well. I am writing in response to the decision regarding the rejection of our manuscript titled " Seasonal Variations in Household Food Insecurity and Consumption Affect Women's Nutritional Status in Rural South Ethiopia " (Manuscript ID: PGPH-D-24-00131) for publication in your Journal. I appreciate the time and effort the editorial board is dedicated to evaluating our work, and I would like to express our gratitude for your valuable feedback. Your insightful comments have helped us identify improvement areas and enhance the clarity of our methodology. We have taken your comment and have made the necessary adjustments to address the concerns raised.

After submitting an application for reconsideration of our rejected manuscript, we received an email from your Journal requesting us to submit the manuscript as a new submission. We have included the Editors' Comments immediately after this letter to show how we addressed the concerns and describe our changes. Editors' comments are shown in bold, and our responses are in plain type. Changes to the manuscript are marked in blue, and the removed text is strikethrough in red in the revised version.

We hope the revised manuscript will better suit your *journal, PLOS Global Public Health.*

Sincerely,

Bethelhem Mezgebe

Hawassa University,

Hawassa, Ethiopia

**REPLY TO THE EDITOR’S COMMENTS**

**Editor’s comment: We recognize that you obtained ethical approval from your local ethics committee after you began the study: your ethics approval document is dated 07 September 2021, while participants were recruited beginning in June 2021. You also state that informed consent was obtained from all participants, which indicates that the samples were prospectively collected for research purposes. For human subjects’ research, PLOS Global Public Health requires that authors obtain ethics committee approval before beginning the study, and we do not accept retrospective ethical approval.**

Response: I apologize for missing an important document regarding ethical approval. I have uploaded the required documentation, which evidenced that data collection was done after obtaining ethical approval. Furthermore, we have provided detailed information in the Ethical Consideration subsection of the manuscript.

Here is some more clarification about the issue;

The submitted manuscript is among my PhD work, conducted under the “Co-producing Gender-responsive Climate Services for Enhanced Food and Nutrition Security and Health in Ethiopia and Tanzania” (COGENT) project. The project has stated in its protocol that Hawassa University staff can utilize the data for their postgraduate degree. However, the University requires PhD students to separately apply for ethical clearance.

The project had already begun data collection on socio-demographics, household food accessibility, household assets, and children’s nutritional status before my involvement in June. The project team received the ethical clearance for the data collection on May 20/20221 (see attached document as other file type (IRB first), with reference number IRB/181/13).

I independently obtained ethical clearance on September 7/2021, for my PhD work with reference number IRB/275/13, and joined working with the COGENT project. I utilized the project's socio-demographic, household food access, and household asset assessment data as baseline data for my research. The time of data collection was mentioned in the manuscript, Method section, data collection subsection, line # 239 -246.

My data include women's body measurements, illness assessment, and level of physical activity, all of which commenced in September 2021.

My research protocol states that the COGENT project funds my research and that I am a member of the group’s research. Thus, for this research, ethical clearance was obtained one month before the start of data collection. The researchers on the first IRB approval (IRB/181/13) include my supervisors and co-author. I have attached the approved protocol. I have added this information to the Method section under the Ethical Consideration subsection of the manuscript (revised manuscript, line #280-292).

Thank you for your attention to this issue, and we look forward to your guidance and feedback on the revised submission.
