# Supplementary material for: Seasonal variations in household food security and consumption affect women’s nutritional status in rural South Ethiopia
Source: PLOS Glob Public Health. 2024 Aug 20;4(8):e0003294. doi: 10.1371/journal.pgph.0003294 (PMC11335107; doi:10.1371/journal.pgph.0003294)
Supplement: Attachment — Submitted filename: response May 10.docx [file pgph.0003294.s004.docx]

Bethelhem Mezgebe , Taye Gari, Mehretu Belayneh, Bernt Lindtjørn

School of Public Health, College of Medicine and Health Sciences, Hawassa University, Hawassa, Ethiopia

[bethelhem_mezgebe@yahoo.com](mailto:bethelhem_mezgebe@yahoo.com)

[tayegari@ymail.com](mailto:tayegari@ymail.com)

[belaynehdinage@gmail.com](mailto:belaynehdinage@gmail.com)

[bernt.lindtjorn@uib.no](mailto:bernt.lindtjorn@uib.no)

Date 10/05/24

PLOS Global Public Health

**Subject:** Revised Manuscript Submission: “Seasonal Variations in Household Food Insecurity and Consumption Affect Women's Nutritional Status in Rural South Ethiopia” (manuscript number PGPH-D-24-00274)

Dear Dr. Dickson,

We are grateful for the opportunity to revise and resubmit our manuscript, “Seasonal Variations in Household Food Insecurity and Consumption Affect Women's Nutritional Status in Rural South Ethiopia,” manuscript number PGPH-D-24-00274, for publication in the PLOS Global Public Health Journal*.*

We have carefully considered all suggestions and concerns and addressed each of them. We utilized the track change feature in word to highlight the modifications made in the manuscript. We have included the Reviewers’ and Editor’s Comments immediately after this letter in the table.

With these comments and revisions, we resubmit the paper.

Sincerely,

Bethelhem Mezgebe

| **Comments of the Editor** | **Response** |
| --- | --- |
| 1. Please amend your detailed Financial Disclosure statement. This is published with the article. It must therefore be completed in full sentences and contain the exact wording you wish to be published.  a. State the initials, alongside each funding source, of each author to receive each grant. b. State what role the funders took in the study. If the funders had no role in your study, please state: “The funders had no role in study design, data collection and analysis, decision to publish, or preparation of the manuscript.” c. If any authors received a salary from any of your funders, please state which authors and which funders.  If you did not receive any funding for this study, please simply state: “The authors received no specific funding for this work. | In response to your suggestion, we have included the financial disclosure statement as follows:  “*The research was funded by the Research Council of Norway (CMI project: 18033). The funding was granted to the Co-producing Gender-responsive Climate Services for Enhanced Food and Nutrition Security and Health in Ethiopia and Tanzania (COGENT) project, which is conducted under Hawassa University. It is important to note that the funders did not have any involvement in the study design, data collection and analysis, decision to publish, or preparation of the manuscript.”* (Revised manuscript, Page 29, line #575-581) |
| **Comments of reviewer # 1** | **Response** |
| Title,  I suggest the authors consider rewriting the objective of the study for clarity. “Food insecurity,” which has a negative connotation, is not consistent with “dietary diversity” and “food consumption” and makes the objective hard to read. The objective can be refined to: “To assess the effect of seasonal variation in household food security, adequate dietary diversity and food consumption, and household income on the nutritional status of women of reproductive age in a rural community in South Ethiopia. Further, the study aimed at identifying associated factors to women's nutritional status | Thank you very much, the comment is accepted, and revision has been made to the title  “*Seasonal variations in household food security and consumption affect women’s nutritional status in rural South Ethiopia.”* (Revised manuscript, Page 0, line #1-3)  We have also revised the objective:  “*To assess the effect of seasonal variation in household food security, adequate dietary diversity, food consumption, and household wealth on the nutritional status of women of reproductive age in a rural community in South Ethiopia. Further, the study aimed at identifying associated factors to women's nutritional status.”* (Revised manuscript, Page 1, line # 28-31 and page 5, line #132-135). |
| Introduction, It would be helpful to the reader if the authors specified in the introduction what information is from Ethiopia and what is from other countries. | Taking your suggestion into account, we have revised the introduction to indicate which information pertains to each country.  See the whole introduction section in the revised manuscript, page 3-6, line #69-137. |
| What was the reason for using “live birth rate” instead of “birth rate”? “Live birth rate is usually used in fertility reporting and not at the population level. | Our intention in utilizing the live birth rate was to show the reproductive health situation of the study area. We have revised the text by substituting the live birth rate with the birth rate.  “*Birth rate 22.8/1000 population”* (Revised manuscript, page 6 line #145) |
| Selection of study population and sample size, Could you add the total number of women included at the end of the section and specify the reason for including all eligible women? | We have carefully considered this point and incorporated it into our revision.  “*The final sample size was estimated to be 904 women. However, to minimize the effect of mobility and the non-respondent rate we include all eligible (1,087 women) in the selected households*.” (Revised manuscript page 9, line #203-205). |
| Wealth index, Line 217: typo. “Cooking” | Following your suggestion, we have thoroughly reviewed the entire manuscript to rectify any grammatical, punctuation, and typographical errors. |
| Comment 6: Meal frequency, I couldn’t find the description of calculating “meal frequency” in the methods. Further, if data is available, including the meal frequency finding in Table 2 would be informative. | We appreciate your comment and addressed it in the materials and methods section.  *“To assess meal frequency, we utilized a 24- hour dietary recall question. Participants were asked yes/no questions regarding the types of meals they had consumed on the previous day during data collection. Based on their responses, the meal frequency was valued as 0: if nothing has been eaten, 1 for one meal/day, 2 for two meals, and 3 for 3 or more meals/day*.” (Revised manuscript, page 11, line #258-262).  Furthermore, we have added an extra row to Table 1, indicating the percentage of women categorized by the type of meal they consumed on the day preceding data collection. (See revised manuscript page 16, line #357). |
| Discussion, I suggest the authors discuss the significance of the findings; the discussion primarily compares the findings of the study with other studies. However, the validity and significance of the findings could have been discussed further. I also would like authors to discuss the implications of funding, not only for future research but also in designing development projects. | Taking your feedback into consideration, we have thoroughly revised our discussion to emphasize the significance and implications of our findings.  We have also re-written our recommendations to ensure they are suitable for informing the design of development plans. (See the discussion section in the revised manuscript, page 21-25, line #416-520). |
| **Comments of reviewer # 2** | **Response** |
| What a new thing your research findings contribute to the science? | We have carefully considered your feedback, and we would like to highlight the following ways in which our findings contribute to the field:   1. One distinct aspect of our study is the utilization of an open cohort design, which enables us to minimize the lost to follow up. (See the revised manuscript, page 6, line # 140, page13, figure 1, and page 25, line #485-494). 2. In our study, we went beyond the common assessment of the wealth index as a one-time measurement; instead, we assessed it three times in a year to capture its temporal variation. (See the revised manuscript, page 12, line # 291-292 and page 17, table 2). Additionally, we examined the effect of seasonal changes in household wealth on the nutritional status of women. We found that in drought-prone areas, under normal circumstances, within a single year, seasonal changes in household wealth have minimal effects on women's BMI, (See the revised manuscript, page 20, table 4). 3. Additionally, we placed emphasis on controlling for the effect of women previous BMI. Our findings revealed that the previous nutritional status has a substantial impact on the current nutritional status. This highlights the importance of considering the longitudinal aspect of nutritional status and the need to account for such conditions when designing interventions. (See the revised manuscript, page 20, table 4). 4. Our findings suggest that food accessibility may be diminished even during the post-harvest season. This has significant implications for intervention efforts, emphasizing the need for interventions not only during pre-harvest seasons but also, in certain cases, during post-harvest seasons, particularly in drought-prone areas. (See the revised manuscript, page 16, table 1 and page 17, Figure 2). |
| The recommendation needs to be revised and in line with the study findings. | Taking your input into careful consideration, we have rewritten the recommendation section to align with our research findings.  (See in abstract, page 1, line # 45-46 and in the recommendation section of the revised manuscript, page 26-28, line #530-568) |
| Use the journal guidelines as all contents of the paper has poor write up | We have thoroughly reviewed the journal's guidelines once again and made efforts to improve our write-up and tables accordingly. |
| Use the scientific tables for the study results |  |
| Your discussion is weak and recommendations also need revision | Taking your suggestions into careful consideration, we have incorporated additional ideas to further enhance the quality and depths of our work. (See the discussion section in the revised manuscript, page 21-25, line #416-520). |
| **Comments of reviewer # 3** | **Response** |
| The Author must include the hypothesis towards the end of Introduction section. | Your input has been instrumental in enhancing the quality of our work. Based on your suggestion, we have included the hypothesis Ho (null hypothesis) in our study, which states: "*Women's body mass index remains the same regardless of seasonal variation in household food security, food consumption, dietary diversity, and wealth*." (Revised manuscript, page 6, line #135-137) |
| The Methods and Materials section is not organised properly and needs to be rearranged. The first subsection must be "Study area description, food production and food availability". The current subsections "Study design" in Line 107 and Study setting and context in Line 110 must be deleted. This means that the content under these deleted subsection will be under new subsection. | Taking your suggestion into careful consideration, we have reorganized the method section based on your comment. In the revised manuscript, the organization of the methods and materials looks at the following   1. Study area description, food production, and food availability 2. Ethical consideration 3. Data collection procedures 4. Data processing and analysis   (See the revised manuscript, Methods and materials section, page 6-14, line #138-323). |
| The subsection "Ethical consideration must be second subsection following Study area description, food production and availability |  |
| The subsection "Variables" in Line 159 must be deleted and be replaced with subsection "Data collection procedures". All the content before Data processing and analysis will be under new subsection of "Data collection procedures" |  |
| It will be interesting for the Author to make recommendation(s) to Policymakers based on this study findings to ensure sustainable nutritious food availability throughout the year including food scarcity seasons. | We have revised our recommendations to incorporate your suggestions, proposing different measures to ensure sustainable year-round food access. (See the recommendation in the abstract and recommendation section in the revised manuscript, page1, line # 45-46, and page, 26-28, line #530-568). |
| There are some minor grammatical errors and the Author needs to go through the manuscript, reading it carefully and correct these. | We have carefully reviewed the entire document and made the necessary corrections to ensure its linguistic accuracy. |
