# Supplementary material for: Seasonal variations in household food security and consumption affect women’s nutritional status in rural South Ethiopia
Source: PLOS Glob Public Health. 2024 Aug 20;4(8):e0003294. doi: 10.1371/journal.pgph.0003294 (PMC11335107; doi:10.1371/journal.pgph.0003294)
Supplement: Attachment — Submitted filename: response to reviewer.docx [file pgph.0003294.s005.docx]

Bethelhem Mezgebe , Taye Gari, Mehretu Belayneh, Bernt Lindtjørn

School of Public Health, College of Medicine and Health Sciences, Hawassa University, Hawassa, Ethiopia

[bethelhem_mezgebe@yahoo.com](mailto:bethelhem_mezgebe@yahoo.com)

[tayegari@ymail.com](mailto:tayegari@ymail.com)

[belaynehdinage@gmail.com](mailto:belaynehdinage@gmail.com)

[bernt.lindtjorn@uib.no](mailto:bernt.lindtjorn@uib.no)

Date17/06/24

PLOS Global Public Health

**Subject:** Revised Manuscript Submission: “Seasonal Variations in Household Food Insecurity and Consumption Affect Women's Nutritional Status in Rural South Ethiopia” (manuscript number PGPH-D-24-00274R1)

Dear Dr. Dickson,

We are grateful for the third opportunity to revise and resubmit our manuscript, “Seasonal Variations in Household Food Insecurity and Consumption Affect Women's Nutritional Status in Rural South Ethiopia,” manuscript number PGPH-D-24-00274R1, for publication in the PLOS Global Public Health Journal*.*

We are deeply grateful to the editor and reviewer for taking the time to provide their expertise and feedback. Their efforts have been invaluable in helping us improve our work. We have carefully considered all suggestions and concerns and addressed each of them. We utilized the track change feature in word to highlight the modifications made in the manuscript. We have included the Reviewer’s and Editor’s Comments in the table.

With these comments and revisions, we resubmit the paper.

Sincerely,

Bethelhem Mezgebe

| **Comments of the Editor** | **Response** |
| --- | --- |
| 1. Please review your reference list to ensure that it is complete and correct. If you have cited papers that have been retracted, please include the rationale for doing so in the manuscript text, or remove these references and replace them with relevant current references. Any changes to the reference list should be mentioned in the rebuttal letter that accompanies your revised manuscript. If you need to cite a retracted article, indicate the article’s retracted status in the References list and also include a citation and full reference for the retraction notice | We have reviewed and checked the reference list for completeness and accurate citation. We also cross-checked the references against the Retraction Watch database and confirmed that none of them have been declared retracted. |
| 2. Please amend your detailed Financial Disclosure statement. This is published with the article. It must therefore be completed in full sentences and contain the exact wording you wish to be published.  a. State the initials, alongside each funding source, of each author to receive each grant. b. State what role the funders took in the study. If the funders had no role in your study, please state: “The funders had no role in study design, data collection and analysis, decision to publish, or preparation of the manuscript.” c. If any authors received a salary from any of your funders, please state which authors and which funders.  If you did not receive any funding for this study, please simply state: “The authors received no specific funding for this work. | In response to your suggestion, we have included the financial disclosure statement as follows:  “*This study was funded by the Research Council of Norway (CMI project: 18033). The funding was granted to the Co-producing Gender-responsive Climate Services for Enhanced Food and Nutrition Security and Health in Ethiopia and Tanzania (COGENT) project conducted under Hawassa University. The funders were not involved in the study design, data collection and analysis, publication decision, or manuscript preparation.”* (Revised manuscript, Page 25, line #491-496) |
| 3. We have noticed that you have uploaded Supporting Information files, but you have not included a list of legends. Please add a full list of legends for your Supporting Information files after the references list. | The list of legends for supplementary table is provided after the reference list.  **“*Supporting information***  ***S1 Table 1: Socio-demographic characteristics of 15 to 49 years old women in South Ethiopia, June 2021****. This table provides the socio-demographic information of 1,089 women participants from 894 households. The data are presented as count (n) and percentage (%). The abbreviation “N” refers to the total number of participants.*  ***S2 Table 2: Socio-demographic characteristics of household heads, South Ethiopia, June 2021****. This table presents the socio-demographic information of the household heads where the study participants reside. The data are presented as count (n) and percentage (%)*.” (Revised manuscript, Page 30, line #711-718) |
| **Comments of reviewer # 1** | **Response** |
| Introduction  The updated introduction section is long and can be rewritten to be more concise. Further, some detailed information about other studies can be used in writing the discussion and not being included in the introduction. | We have shortened the introduction by excluding detailed information. (Revised manuscript, Page 2-4 , line #50-98) |
| Could you please check that "paid activities" L88 refers to paid and not unpaid? | We have rewritten it. It is to mean that rural women involve in certain income generating activities particularly in lean period. (Revised manuscript, Page 2, line #59) |
| L285, I believe taking height measures in adults for two times addresses reliability or accuracy of measures and not validity. | The statement has been revised as *“To improve the data accuracy, the height of the women was taken in two season.\|*” (Revised manuscript, Page 11 , line #247) |
| Did you use dietary recall to assess food consumption? If so and you haven't mentioned it somewhere, could you add it? | "We used a food frequency questionnaire to assess food consumption. The questionnaire consisted of eight response options that enabled the respondents to recall how frequently they ate particular food groups.  The response options were:   1. *Once per week* 2. *Twice per week* 3. *3 times per week* 4. *4 times per week* 5. *2 or more times per month* 6. *Once per month* 7. *Yearly* 8. *Never*   We have incorporated this list of response options in the revised manuscript, which clarifies the use of the dietary recall method.  (Revised manuscript, Page 8, line #185 - 189) |
| Table 3: numbers don't add up. Please check and if this is because of missing data, note that. | Based on your feedback, we have made the following revisions:  In the subtitle for "meal type" in Table 1, the categories were previously not mutually exclusive, which is why they did not sum up to the total observation of 1,040. We have now presented the meal type categories in an exclusive manner so that they do sum up to the total 1,040 observations (100%).  However we have removed figure 3 since it conveys similar information to the subtitle of table regarding meal type.  Additionally, we have thoroughly rechecked the sums in all of the tables and have corrected any discrepancies we found. (See the revised manuscript, page 15, table 1). |
| Discussion: -Generally, before comparing the findings of the study to other studies, the importance and significance of the findings should be discussed. How accurate and relevant are the findings? I recommend re-writing the discussion.  - What does "weighted" food consumption mean? - Regarding PSNP, when was the BMI of beneficiaries were lower than their peers? I wonder if the recipients of PSNP had lower BMI than others and that's the reason they were part of the program. - Please check L481: is there any evidence to suggest the impact of changes in seasonal wealth on women's nutrition status? -L503: What does information bias include? | We rewritten the discussion based on your suggestion.  We utilize the term “weighted food consumption” to indicate that the food consumption score was calculated by weighting the weekly food group consumption by its nutrient density. (Revised manuscript, Page 8, line #190-197). We have cancelled the term weighted and consistently used food consumption.  Regarding the Productive Safety Net Program (PSNP), the study found that the BMI of PSNP beneficiaries was lower than their counterparts in all seasons. This suggests an initial difference in average BMI between PSNP beneficiaries and non-beneficiaries. Therefore, in this study, it may not be appropriate to draw conclusions about the program's effect on women's nutrition. We suggest that further investigation is needed by other researchers to better understand the impact of the PSNP on women's nutritional outcomes. (Revised manuscript, Page 23 & 24, line #454-461).  Regarding the evidence on household wealth, we did not find direct evidence to suggest the impact of changes in seasonal wealth on women's nutritional status. This is because most studies assess household wealth at a single point in time, lacking the temporal relationship. However, these studies generally show a positive relationship between household wealth and women's nutritional status. Additionally, many studies use seasonal variations in household food security, dietary diversity, and food consumption as indirect measures of seasonal changes in household economy.  Since we lacked direct evidence on the relationship between seasonal wealth changes and women's nutrition, we have decided to remove this idea from the revised manuscript. (Revised manuscript, Page 20, line #379 & 380).  Information bias refers to any erroneous information that arises during the data collection process, including interviewing and anthropometric measurements. It includes recall bias, social desirability bias, and intrapersonal and interpersonal measurement variability and so on. |
| Recommendation: What is the difference between year-round access and sustainable access? I recommend merge these two categories and group the recommendations under different stakeholders such as policy makers, local government, etc. | We used the term "year-round access" to indicate that there is adequate food available in all seasons within a year. The term "sustainability" is used to mean avoiding seasonal food shortages over the long term or permanently.  We have restructured the recommendation according to your feedback. (Revised manuscript, Page 24, line #475-483). |
| I recommend a final edit of the document for English language to prepare a concise version. | We have made a thorough edit to improve the English language and shorten the overall length. You can review the revised manuscript with track changes so you can see the specific edits that have been made. |
